# Marine Natural Products with P-Glycoprotein Inhibitor Properties

**DOI:** 10.3390/md12010525

**Published:** 2014-01-22

**Authors:** Dioxelis Lopez, Sergio Martinez-Luis

**Affiliations:** 1 Center for Drug Discovery and Biodiversity, Institute for Scientific Research and Technology Services (INDICASAT), Clayton, City of Knowledge, P.O. Box 0843-01103, Panama; E-Mail: dioxelis.lopez@indicasat.org.pa; 2 Department of Biotechnology, Acharya Nagarjuna University, Nagarjuna Nagar, Guntur 522510, India

**Keywords:** P-glycoprotein, marine natural products, multidrug resistance

## Abstract

P-glycoprotein (P-gp) is a protein belonging to the ATP-binding cassette (ABC) transporters superfamily that has clinical relevance due to its role in drug metabolism and multi-drug resistance (MDR) in several human pathogens and diseases. P-gp is a major cause of drug resistance in cancer, parasitic diseases, epilepsy and other disorders. This review article aims to summarize the research findings on the marine natural products with P-glycoprotein inhibitor properties. Natural compounds that modulate P-gp offer great possibilities for semi-synthetic modification to create new drugs and are valuable research tools to understand the function of complex ABC transporters.

## 1. Introduction

### 1.1. P-Glycoprotein

P-glycoprotein (P-gp), belonging to the large ATP-binding cassette (ABC) family, is a transmembrane protein encoded by the ATP-binding cassette sub-family B member 1 (ABCB1) gene. This protein is composed of 1280 amino acids (170 kDa) organized in two transmembrane domains, each one comprised of twelve highly hydrophobic α-helices and two intracellular nucleotide binding regions with ATPase activity. The drug-binding site (DBS) is found in the intracellular part of the protein, and when ATP activates P-gp, the substrate is extruded by a “flip-flop” mechanism to the luminal side. Its subsequent dephosphorylation leads to the transformation of the protein back to the initial state [1,2,3,4].

### 1.2. Functions of P-gp

P-gp can transport a wide range of xenobiotics out of the cell at the apical membrane of many secretory cell types, including adrenal gland, brain, kidney, liver, placenta, small and large intestine and the testes. Not all functions of the P-gp are known; however, there is a growing understanding of the role of P-gp in many organisms [1,2,3,4,5,6,7,8,9,10].

In the blood-brain barrier and blood-placenta barrier, P-gp prevents xenobiotic accumulation in the brain and pregnant uterus, respectively. Usually, P-gp excretes xenobiotics that are taken along with nutrients through the urine, bile and intestinal lumen and translocates hormones. The expression of P-gp in normal gastrointestinal tract cells prevents drug absorption after oral administration. Similarly, P-gp in the brain blocks the entrance of antiviral drugs [5,6,7,8,9,10].

### 1.3. Mechanism of P-gp Efflux Function

**Figure 1:**
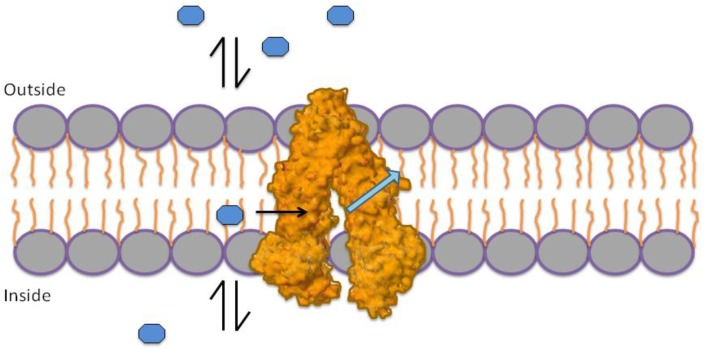
Flippase mechanism. P-gp interchanges molecules from the inner leaflet of the membrane to the outer leaflet, in order to maintain a concentration balance on both sides.

The mechanism by which P-gp performs its efflux pump function is not completely understood; however, there are several models to explain this process, two of which are most accepted. The first model states that P-gp exerts its effect through a flippase type mechanism (Figure 1) [11,12]. This mechanism is based on the assumption that drugs in the external and internal medium of the membrane should be at equilibrium with the outer and inner leaflet of the membrane. Based on this equilibrium, P-gp exchanges drugs from the inner leaflet of the membrane to the outer leaflet [11].

**Figure 2:**
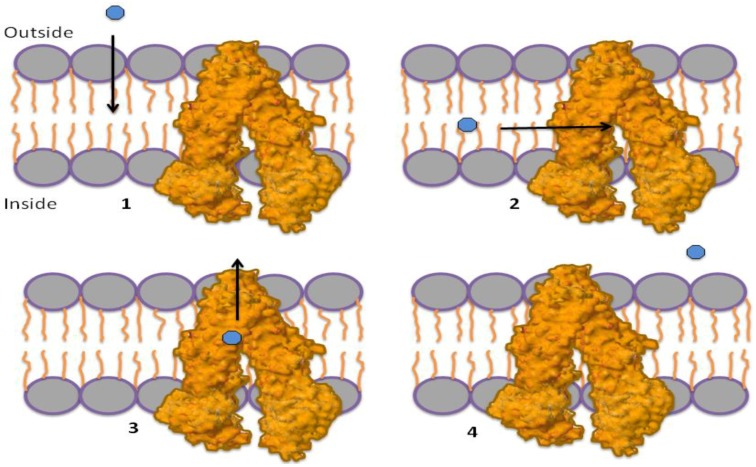
Hydrophobic vacuum cleaner mechanism. (**1**) Substrates pass through the membrane to the lipid bilayer; (**2**) substrates can enter the P-gp through “portals” that pass the substrate from the lipid bilayer in to the P-gp internal cavity; (**3**) substrates bind to the drug-binding site (DBS); (**4**) P-gp transports the substrate to the outside of the cell.

Conversely, the “hydrophobic vacuum cleaner” model (currently the most accepted) suggests that P-gp removes hydrophobic molecules that are within in the membrane by a “vacuum cleaner” device (Figure 2) [12,13,14]. The high-resolution crystal structure of a mouse P-gp does not differentiate between the models, but rather, could be used to support either of them. The protein structure contains two portals within the P-gp cavity that connect to the inner leaflet of the membrane and would allow hydrophobic drugs to pass directly from the lipid bilayer to the cavity [14].

### 1.4. Role of ATP in Protein Activation

In the absence of exogenous substrates, P-gp has an intrinsic ATPase activity [15]. Moreover, P-gp substrates can positively or negatively modulate this activity and, consequently, the rate of hydrolysis of ATP [16]. Compounds that modulate the P-gp ATPase activity have been categorized into three groups: (1) Compounds that stimulate basal ATPase activity at low concentrations and inhibit the activity at high concentrations (e.g., vinblastine, verapamil and paclitaxel); (2) compounds that increase ATPase activity in a dose-dependent manner (e.g., bisantrene, valinomycin and tetraphenylphosphonium); and (3) compounds that inhibit both basal and compound-stimulated ATPase activity (e.g., cyclosporin A, rapamycin and gramicidin D) [16].

A particularly important aspect to consider in the mechanism of P-gp efflux function is the role of ATP in protein activation. Higgins and coworkers proposed that two molecules of ATP are required in the first step of protein activation [17]. Siarheyeva and colleagues suggested a mechanism that also involves two molecules of ATP, but one ATP molecule is strongly bound and the other weakly bound to the protein, affording two specific substrate-binding domains, one of high affinity and one of low affinity [18]. Sauna and coworkers proposed another model in which one molecule of ATP activates the efflux pump of P-gp to move drugs out of the protein, and a second ATP molecule returns P-gp to its original conformation [19].

All these models predict that inhibiting the protein ATPase activity can disrupt P-gp efflux function. However, this is not a general rule, because some P-gp inhibitors increase the protein ATPase activity [20,21,22,23]. Furthermore, initial investigations have suggested that P-gp inhibitors should bind to the protein nucleotide binding site (NBS) to inhibit ATPase activity, but one study of a flavonoid-type inhibitor found that the compound stimulated the activity of ATPase without binding to the protein NBS [22].

In general, P-gp inhibitors work in one of the three ways:

1. Promoting a conformational change in P-gp that blocks the ATP binding site and, subsequently, ATPase function;
2. Promoting a conformational change in P-gp that enhances ATP binding, but concurrently blocks the substrate binding site;
3. Inactivating the substrate binding site without inducing any conformational changes, e.g., stereo-isomers of cyclic hexapeptide inhibitors QZ59-RRR and QZ59-SSS [24].

### 1.5. Importance in Therapy

Due to its role in drug metabolism, P-gp has considerable clinical relevance; it affects the absorption, distribution and secretion of drugs, and it has a major role in multidrug resistance (MDR) in cancer [4,5,6,7,8,9]. P-gp reduces the clinical efficacy of several drugs (anticancer, antibiotics, antidepressants, antiepileptics, antihistamines, antihypertensives, antiarrhythmics, calcium channel blockers, cardiac glycosides, immunosuppressants, HIV protease inhibitors, hypocholesterolaemiants and steroids) by modifying their absorption and distribution in tissues [1,2,6,8,9,10].

P-gp was first discovered in 1976 for its role in MDR in cancer; it is overexpressed in several human tumors and is an important barrier to success in cancer treatments [3,4,5,6,7,8,10]. Interestingly, P-gp in tumors also appears to provide resistance to apoptosis induced by different stimuli, including serum starvation, Fas, TNF and UVB- and γ-irradiation [5,8]. The mechanism whereby P-gp inhibits apoptosis is still unclear [5,8].

In AIDS patients, P-gp contributes to the resistance to protease inhibitors, such as indinavir, ritonavir, nelfinavir and saquinavir [2,25]. P-gp is also involved in MDR in some human parasitic infections, including those caused by *Plasmodium falciparum* [26], *Leishmania tropica* [27], *Leishmania amazonensis* [28], *Trypanosoma cruzi* [29] and *Entamoeba histolytica* [30].

### 1.6. P-gp Inhibitors

Over the years, identifying small molecules that interfere with the activity of P-gp has taken relevance, because blocking its pump function could reduce the effective concentration of drugs administered in the treatment of cancer, HIV, parasitic diseases and other diseases. On the basis of their specificity, affinity and toxicity, P-gp inhibitors are categorized into three generations (Table 1). The first generation of inhibitors are metabolites that already have a clinical use, e.g., verapamil (calcium channel blocker drug) and cyclosporine A (immunosuppressant drug), and, subsequently, were tested against P-gp and found to inhibit the enzyme. These drugs required high concentrations to inhibit P-gp, and for this reason, they were not approved as inhibitor P-gp drugs [31,32].

Second generation inhibitors are compounds without previous therapeutic use and that have a higher affinity for P-gp than the first generation compounds. The problem with these metabolites is that they are quickly metabolized by the CYPA4 enzyme, thus altering their pharmacokinetics and reducing their efficacy. It is important to point out that these inhibitors are designed to have lower toxicity than the compounds belonging to the first generation, despite retaining certain undesirable toxicity features that limit their pharmacological use [33,34,35].

**Table 1:** Selected examples of classical inhibitors of P-gp by generation.

First Generation	Second generation	Third Generation
Verapamil Cyclosporine A Vincristine Reserpine Quinidine Tamoxifen Trifluoperazine	(*R*)-verapamil Valspodar (PSC-833) Dexniguldipine Elacridar (GF120918) Biricodar Dofequidar	Tariquidar (XR9576) Zosuquidar (LY335979) Laniquidar (R101933) ONT-093 (OC-144-093) Mitotane (NSC-38721) Annamycin

The third generation inhibitors are compounds that were obtained using combinatorial chemistry and subsequent structure-activity relationship studies to identify compounds that inhibit P-gp with high specificity and low toxicity. These P-gp inhibitors have a potency of about 10-fold more than the earlier generations of inhibitors. These compounds are not inhibited by the CYPA4 enzyme, and therefore, they do not exhibit altered pharmacokinetics [36,37].

Inhibitors belonging to one of the three generations exert their effect by one of the following mechanisms (Table 2): (1) Disrupting the hydrolysis of ATP [38,39,40,41,42,43,44,45,46,47]; (2) altering P-gp expression [48,49,50,51,52,53,54,55]; and (3) reversible inhibition or competition for a binding site, as demonstrated by photoaffinity labelling [56,57,58,59,60,61,62,63,64,65].

**Table 2:** Mechanism of P-gp classical inhibitors.

ATPase Activity		P-gp Expression		Competition for Binding Site
Inhibitor	Stimulator	Down Regulator	Up Regulator	
Valspodar Tariquidar Elacridar ONT-093	Verapamil Cyclosporine A Vincristine Quinidine Tamoxifen Toremifene Trifluoperazine Dexverapamil Biricodar	Verapamil Cyclosporine A Reserpine Toremifene Trifluoperazine Dexverapamil Valspodar	Vincristine	Verapamil Cyclosporine A Vincristine Reserpine Quinidine Valspodar Dexniguldipine Biricodar Elacridar Dofequidar Tariquidar Zosuquidar

One of the most common mechanisms displayed by classical P-gp inhibitors is competition for drug binding sites. However, P-gp has multiple binding sites, making it difficult to design target-based inhibitors. The poor selectivity shown by inhibitors with this mechanism could be due to P-gp having multiple binding sites. It is also difficult to detect general functional groups that modulate inhibitory activity against P-gp; however, it has been possible to obtain active functional groups from specific pharmacophores.

**Table 3:** P-gp inhibitors that have been evaluated in clinical trials.

P-gp Inhibitor	Phase	Trial	Protocols Identification
Tariquidar (XR9576)	II	Tariquidar and Docetaxel to Treat Patients With Lung, Ovarian, Renal and Cervical Cancer	03-C-0284, NCI-03-C-0284, NCT00072202, NCT00069160
	II	Surgery Plus Chemotherapy (Doxorubicin, Vincristine and Etoposide), Mitotane and Tariquidar to Treat Adrenocortical Cancer	040011, 04-C-0011, NCT00071058
	I	Study of XR9576 and Vinorelbine in Patients with Advanced Cancer	NCI-00-C-0044
	I	Trial of Tariquidar (XR9576) in Combination with Doxorubicin, Vinorelbine or Docetaxel in Pediatric Patients with Solid Tumors	NCT00011414
Zosuquidar (LY335979)	III	Daunorubicin and Cytarabine ± Zosuquidar in Treating Older Patients with Newly Diagnosed Acute Myeloid Leukemia or Refractory Anemia	CDR0000257122 E3999, U10CA021115, ECOG-E3999, NCT00046930
	II	Zosuquidar in Combination With Daunorubicin and Cytarabine in Patients Ages 55–75 with Newly Diagnosed Acute Myeloid Leukemia (AML)	KAN-979-01 NCT00129168
	II	A Trial of Gemtuzumab Ozogamicin (GO) in Combination with Zosuquidar in Patients with CD33 Positive Acute Myeloid Leukemia	KAN-979-02 NCT00233909
Laniquidar (R101933)	II	R101933 Combined with Chemotherapy in Treating Patients with Metastatic Breast Cancer That Has Not Responded to Previous Chemotherapy	EORTC-10003-16004 EORTC-16004, ECSG-EORTC-16004, IDBBC-10003, NCT00028873
Elacridar (GF120918)	I	A Phase I, Randomized, Open-Label, Parallel-Cohort, Dose-Finding Study of Elacridar (GF120918) in Combination with 2.0 mg Oral Topotecan in Cancer Patients	BCR10001
Mitotane (NSC-38721)	III	Trial in Locally Advanced and Metastatic Adrenocortical Carcinoma Treatment (FIRM-ACT)	CO-ACT-001 NCT00094497
	II	Phase II Study of Continuous-Infusion DOX/VCR/VP-16 with Daily Oral Mitotane for Renal Cell Cancer	NCI-94-C-0156
	II	Phase II Mitotane plus Cortisone Acetate/Fludrocortisone and ADR for Residual, Recurrent or Metastatic Adrenal Cortical Carcinoma	EST-1879
	II	Phase II Study of Continuous-Infusion DOX/VCR/VP-16 with Daily Oral Mitotane Before and After Surgery in Patients with Adrenocortical Carcinoma	NCI-93-C-0200D NCI-93-C-0200B
Annamycin	II	Chemotherapy in Treating Patients with Breast Cancer	CDR0000068486 NYU-9851, NCI-G01-1914, NCT00012129

FIRM-ACT: First International Randomized trial in locally advanced and Metastatic Adrenocortical Carcinoma Treatment; DOX: Doxorubicin; VCR: Vincristine; VP-16: Etoposide; ADR: Adriamycin.

One of the most interesting studies focused on identifying functional groups of active molecules, which was performed with 27 digoxin transport inhibitors in Caco-2 cells, found that two hydrophobic groups along with a hydrogen-bond acceptor group and an aromatic core were required for P-gp inhibition [66] This finding is relevant, because many previously reported P-gp inhibitors also possesses the majority of these features. It is also important to highlight that peptides similar to cyclosporine A (in size and amino acid composition) could have the inhibitory properties of P-gp, as in the case of valspodar and kendarimide A.

Many inhibitors of P-gp, especially within the third generation compounds, have been tested in clinical trials to assess their pharmacological potential (Table 3). Unfortunately, most of them have failed, because they displayed non-specific toxicity [8]. Among the negative factors that prevented success are: (1) High variability in the response rate associated with P-gp inhibitors, which is related to levels of P-gp expression and the co-expression of other ABC transporters [1,8,67]; (2) the pharmacokinetic interaction between the P-gp inhibitor and the other co-administered drugs, which lead to an increase in drug toxicity [8]; (3) the increase in plasma concentrations of a co-administered drug by interfering with its metabolism or excretion [8]; and (4) the increase in the toxicity of a co-administered drug in healthy tissues by inhibiting the basal activity of P-gp [8]. Therefore, there is an urgent need for identifying new, more effective and non-toxic P-gp inhibitors.

## 2. Inhibitors from Marine Sources

Oceans cover around seventy percent of the Earth’s surface and represent a resource of huge dimensions for natural product chemistry. This media contains nearly eighty percent of the biological diversity of life on the planet. Studies over the past 30 years have led to the discovery of thousands of new compounds from marine sources, which have shown a wide range of biological activities [68,69,70].

Several marine compounds or analogs inspired by marine natural products have been approved for clinical use, including vidarabine (for the treatment of a recurrent epithelial keratitis caused by herpes simplex virus type 1 and 2 and superficial keratitis), cytarabine (for cancer), ziconotide (for the treatment of severe chronic pain in patients with cancer or AIDS), trabectedin (for use as an anticancer agent against soft tissue sarcoma) and halaven (for metastatic breast cancer) [68,69,70].

In this article, we systematically review several marine natural products with P-gp inhibitor properties (Table 4). As mentioned, this protein is a major cause of drug resistance in cancer, some parasitic diseases, epilepsy and other disorders. The modulation of targets of P-gp by natural or synthetic compounds offers great possibilities for the discovery of new drugs and valuable research tools to understand the complex ABC transporters.

**Table 4:** Marine compounds with P-gp inhibitor properties. ET-743, ecteinascidin 743; MDR, multi-drug resistance.

Inhibitor	Intracellular Accumulation of Substrates	ATPase Activity	Photoaffinity Labelling	Cell Line Tested	Drug with Enhanced Activity	P-gp Expression	Selective to MDR1 or ABCB1
Sipholenol A	Increased	Stimulated	Inhibited	KB-C2, KB-V1	colchicine, vinblastine, paclitaxel	Not altered	Yes
Lamellarin	Increased	xx	xx	P338/Schabel	doxorubicin, daunorubicin, vinblastine	xx	xx
Agosterol A	xx	xx	Inhibited	KB-C2	colchicine	xx	No
ET-743 ^+^	Increased	xx	Not inhibited.	KB-8-5, KB-C2	doxorubicin, vincristine	Downregulated	xx
*N*-Methylwelwitin-dolinone C isothiocyanate	Increased	xx	Inhibited	NCI/ADR-RES	vinblastine, taxol, actinomycin D, daunomycin, colchicine	xx	xx
Parguerenes	Increased	xx	xx	SW620AD-300, HEK293/ABCB1, CEM/VLB100	vinblastine, doxorubicin and paclitaxel	Not altered	No
Patellamide d	xx	xx	xx	CEM/VLB100	vinblastine, colchicine and adriamycin	xx	xx
Kendarimide A	xx	xx	xx	KB-C2	colchicine	xx	xx
Bryostatin 1	Increased	xx	Inhibited	KB-C1, HeLa-MDR1-V185	vinblastine, colchicine		xx
ISA, ISA B	xx	xx	xx	KB/VJ300	vincristine	xx	xx
Nocardioazines	xx	xx	xx	SW620AD-300	doxorubicin	xx	xx
Discodermolide *	xx	xx	xx	SW620AD-300, A2780AD	xx	xx	No
Polyoxygenated steroids ^#^	xx	xx	xx	KB-C2	xx	xx	xx

^+^ Inhibits the expression of MDR1; * the authors expressed only a reduction in resistance to paclitaxel; ^#^ the authors expressed only an inhibition in the growth of MDR cells; xx: Information not reported; P388/Shabel: MDR murine leukemia cells; HEK293/ABCB1: Human primary embryonic kidney stable gene-transfected cell line.

### 2.1. Inhibitors from Tunicates

**Figure 3:**
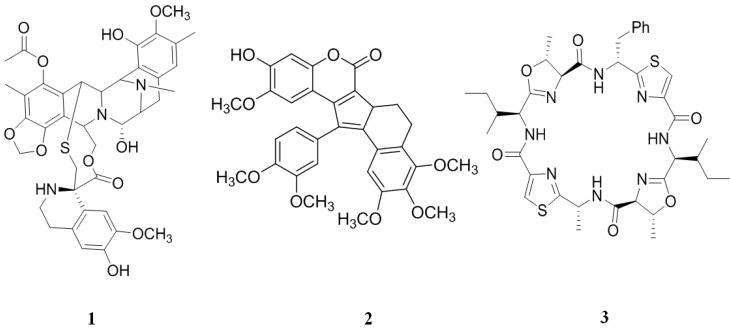
Inhibitors of P-gp that have been isolated from tunicates.

The compound, ecteinascidin 743 (ET-743, **1**) (Figure 3), isolated from the Caribbean tunicate, *Ecteinascidia turbinata* [71,72], showed good anti-cancer *in vitro* activity against mouse lymphocytic leukemia (L1210) cells with a half maximal inhibitory concentration (IC_50_) values of 0.5 ng/mL. ET-743 partially reverses resistance to doxorubicin and vincristine in MDR epidermal carcinoma (KB-C2 and KB-8-5) P-gp/multidrug resistance 1 (MDR1) overexpressing cancer cell lines. A greater intracellular accumulation of doxorubicin and vincristine (up to 122 and 22 fold, respectively) were observed in both cells when pretreated with non-toxic concentrations of **1**. However, photoaffinity labeling experiments showed that overcoming doxorubicin/vincristine resistance was not a result of the direct inhibition of P-gp activity [73]. Because of these beneficial effects in cancer treatments, **1** has received orphan drug designation specifically for soft tissue sarcoma treatment in the United States and ovarian cancer treatment in the United States and Europe [74].

Lamellarins are a group of polyaromatic alkaloids originally isolated from *Lamellaria* sp. [75] and later from the ascidian, *D. chartaceum* [76,77], the sponge, *Dendrilla cactos* [78,79], and some species of unidentified ascidians [80,81,82]. This class of compounds has shown diverse biological activities, including cytotoxicity [75,83,84], immunomodulating activity [77], inhibition of HIV integrase [83] and, critically, the ability to render some MDR cancer cell lines susceptible to anti-cancer treatments [84].

Lamellarin I (**2**) (Figure 3) presented a better chemo-sensitizing activity than verapamil (nine to 16 fold higher) in doxorubicin-resistant human colon adenocarcinoma (Lo Vo/Dx) cell line. In addition, **2** increases the cytotoxicity of doxorubin, vinblastine and daunorubicin in a concentration-dependent manner in MDR cells. Compound **2** exerts this effect through a direct inhibition of the P-gp pump function, as demonstrated by the accumulation of Rhodamine 123 in Lo Vo/Dx cells [84].

The patellamides are thiazole- and oxazoline-containing cyclic octapeptides isolated from *Lissoclinum patella* that show several biological activities, including cytotoxicity and reversing resistance in the MDR human leukemic (CEM/VLB100) cell line against vinblastine, colchicine and adriamycin [85,86]. The cytotoxicity of patellamide-type compounds may be because of conformational restrictions set by the presence of the heterocycles and their ability to intercalate DNA [86]. Of this family of compounds, patellamide D (**3**) (Figure 3) showed the best activity in reversing MDR; it enhanced by 66, 2.8 and 1.4 fold the activity of vinblastine, adriamycin and colchicine, respectively. The activity of **3** is similar to verapamil, a well-known P-gp inhibitor [87].

### 2.2. Inhibitors from Sponge

**Figure 4:**
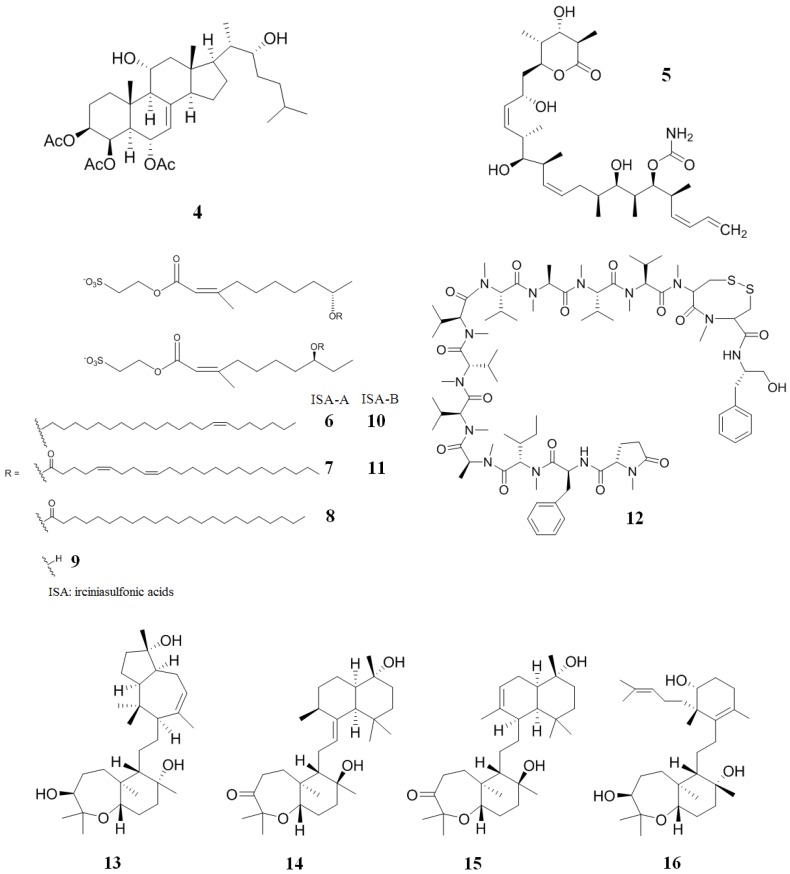
Inhibitors of P-gp that have been isolated from sponges.

A novel polyhydroxylated sterol acetate, agosterol A (**4**) (Figure 4), was isolated from the marine sponge, *Spongia* sp. [88]. This compound completely reversed MDR in human KB carcinoma cells overexpressing an MRP1 (a membrane glycoprotein) [88,89]. In order to obtain the mechanism of action of **4**, accumulation and efflux experiments were performed using KB-C2 and human carcinoma overexpressing MRP1 (KB-CV60) cell lines [89]. Compound **4** interrupted the ATP-dependent active efflux of vincristine in both cells by increasing intracellular concentrations of this Vinca alkaloid. In other experiments, **4** inhibited the [^3^*H*] azidopine photolabeling of P-gp and the uptake of [^3^*H*]*S*-(2,4-dinitrophenyl)glutathione in inside-out membrane vesicles from KB-CV60 cells [90]. Taken together, the data indicate that **4** inhibits the drug efflux modulated by P-gp and MRP1.

Discodermolide (**5**) (Figure 4) is a polyketide that was discovered in the marine sponge, *Discodermia dissoluta*, due to its immunosuppressive and anti-tumor activities [91,92,93]. Interestingly, **5** has the same mechanism of action as taxol (it blocks the cell cycle at the G"/M checkpoint and induces apoptosis), but is more potent against several types of cancer cell [94]. Additionally, **5** is active against taxol-resistant cells. Normally, MDR ovarian carcinoma (A2780AD) and paclitaxel-resistant colon carcinoma (SW620AD-300) cell lines are 25–89 fold more drug-resistant than their parenteral lines. Compound **5** drastically decreased multidrug resistance to taxol in both cell lines, with IC_50_ values in the range of 70 nM for SW620AD-300 (compared to 260 nM for taxol) and 580 nM for A2780AD cells (compared to 3900 nM for taxol) [94].

A mixture of three structurally-related esters (irciniasulfonic acids, **6**–**11**) (Figure 4) were isolated from the marine sponge *Ircinia* sp. The irciniasulfonic acids mixture are found to reverse MDR at 33 µg/mL against P-gp overexpressing derived from human cancer KB cells (KB/VJ300) cells in the presence of 10 ng/mL of vincristine. It was subsequently discovered that a simple chain of deacylirciniasulfonic acid (**9**) (Figure 4) was 13 fold more potent than the other irciniasulfonic acids (IC_50_ values of 3 μM and 38 μM, respectively) [95,96].

Kendarimide A (**12**) (Figure 4), isolated from the marine sponge, *Haliclona* sp., reversed multidrug resistance in a human carcinoma cell line overexpressing P-gp [97]. Compound **12** completely reversed the resistance to colchicine in KB-C2 cells at a 6 μM concentration. A mixture of 6 µM of **12** and 0.1 µg/mL of colchicine inhibited the growth of KB-C2 cells by 87%, while **12** showed no inhibitory activity against human epidermoid carcinoma (KB-3-1) cells at a 6 µM concentration. It is important to point out that cyclosporin A, a potent peptide with activity against multi-drug resistant P-gp, is composed of a similar number of amino acid residues as **12**. This might indicate that peptides having an analogous amino acid length have the ability to reverse multidrug resistance [97].

Sipholenol A (**13**) (Figure 4) is a triterpenoid isolated from the Red Sea sponge, *Callyspongia siphonella* [98]. This sponge is a prolific producer of about 30 different triterpenes grouped into four major families: Sipholane, siphonellane, neviotane and dahabane. The sipholane family has the ability to reverse P-gp-mediated MDR in some cancer cells [98,99]. Compound **13** enhanced the cytotoxicity of three well-known P-gp substrates (colchicines, vinblastine and paclitaxel) in KB-C2 and MDR human cervix carcinoma subclone derived from KB-3-1 (KB-V1) cells. In this experiment, **13** decreased the multidrug resistance of these cells in a concentration-dependent manner [100]. Finally, it was found that **13** increases the accumulation of paclitaxel by inhibiting the P-gp efflux function, stimulates ATPase activity, inhibits the photolabeling of P-gp using [125*I*]-iodoarylazidoprazosin as the transport substrate and does not affect P-gp expression. Further research demonstrated that sipholenone E (**14**), sipholenol L (**15**) and siphonellinol D (**16**) (Figure 4) showed similar activity to **13** [101]. 

### 2.3. Inhibitors from Cyanobacteria and Alga

**Figure 5:**
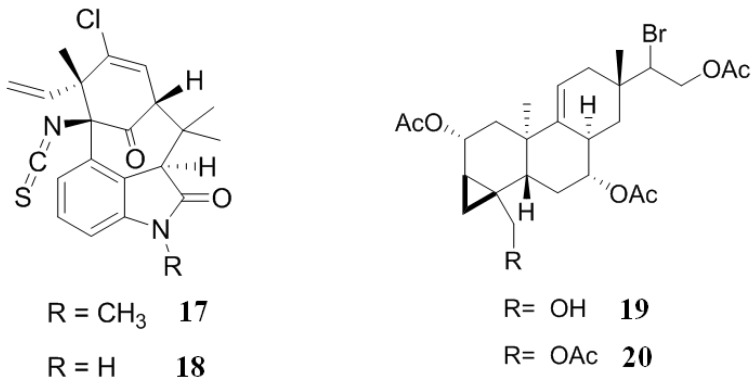
Inhibitors of P-gp that have been isolated from cyanobacteria and algae.

Welwitindolinones are a group of alkaloids isolated from the cyanobacteria, *Hapalosiphon welwitschii*. *N*-Methylwelwitindolinone C isothiocyanate (**17**) (Figure 5) enhanced the cytotoxicity of two anticancer drugs, actinomycin D and daunomycin, in vinblastine-resistant ovarian carcinoma (SK-VLB-1) cell lines. Additionally, **17** increased the activity of anticancer drugs vinblastine, taxol, actinomycin D, colchicine and daunomycin in MDR ovarian adenocarcinoma (NCI/ADR-RES) cell lines, formerly known as MDR breast carcinoma (MCF-7/ADR) cells. Another member of this alkaloid family, welwitindolinone C isothiocyanate (**18**) (Figure 5), presented a weak activity, indicating that the methyl group is important for biological activity. Moreover, when an isonitrile group replaced the isothiocyanate group, the compound was inactive, demonstrating that the isothiocyanate group is also important for the biological activity shown by these alkaloids [102].

Two unusual brominated diterpenes, parguerenes I (**19**) and II (**20**) (Figure 5), obtained from the Australian marine red alga, *Laurencia filiformis*, are non-cytotoxic inhibitors of P-gp and MRP1 mediated drug efflux. These two small molecules exhibited a dose-dependent reversal of P-gp mediated efflux of vinblastine, doxorubicin and paclitaxel MDR, without altering the levels expression of P-gp. This suggests that **19** and **20** inhibit P-gp efflux by a novel mechanism of action or at least by a mechanism this is very different to other known inhibitors, such as verapamil and cyclosporin A, due to **19** and **20** interacting with and upsetting the extracellular antibody binding epitope of P-gp. A detailed analysis of the structure-activity relationship between **19** and **20** indicates that it is possible to manipulate and optimize the core pharmacophore of these molecules in order to increase their activity [103].

### 2.4. Inhibitors from Bryozoans

**Figure 6:**
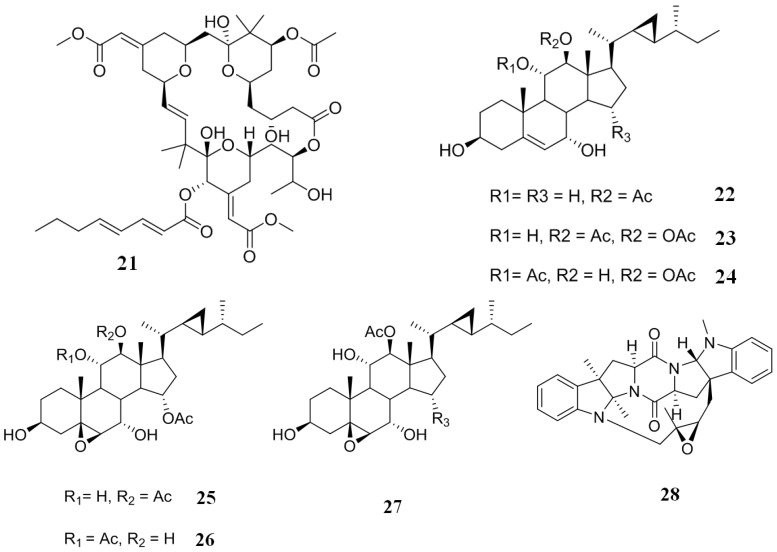
Inhibitors of P-gp that have been isolated from bryozoans, corals and marine bacteria.

From a species of bryozoan, *Bugula neritina*, have been isolated a group of macrolide lactones called bryostatins. These compounds are potent modulators of protein kinase C, and they are anticancer and memory enhancing agents [104,105]. Probably the most important member of this compounds family is bryostatin 1 (**21**) (Figure 6), which is a modulator of protein kinase C with a potency similar to that of the tumor-promoting phorbol ester, 12-*O*-tetradecanoylphorbol-13-acetate (TPA) [106,107]. Compound **21** also has the ability to modulate the P-gp mediated MDR. In an experiment using two cell lines overexpressing a mutant MDR1 encoded P-gp, colchicine-resistant MDR clone of KB cells (KB-C1) and human epitheloid cervix carcinoma (HeLa) cells transfected with an MDR1-V185 build (HeLa-MDR1-V185) that contains a valine instead of a glycine in position 185, it is found that **21** reversed the resistance developed against vinblastine and colchicine by both cells [108]. Additionally, **21** is capable of reversing P-gp mediated MDR in HeLa cells transfected with MDR1-V185 by an increase of the intracellular accumulation of rhodamine 123. From these experiments, it was evident that **21** joins to sites G185 and V185 P-gp, and thus, this compound is able to reverse a mutant P-gp specifically [108]. 

### 2.5. Inhibitors from Corals

The octocoral, *Isis hippuris*, is a prolific producer of several polyoxygenated steroids, including gorgosterol, hippuristanol, hippuristerone and hippuristerol types [109]. A few of the first type have been tested against KB-C2 overexpressing P-gp and against KB-CV60 overexpressing multidrug resistance protein-1 cells. Most steroids (**22**–**27**) (Figure 6) displayed moderate activity against KB-C2 cells (a percentage growth inhibition of 68.0, 70.0, 87.8, 89.2, 87.4 and 82.1, respectively, at a concentration of 3 µg/mL), but not against KB-CV60 cells (a percentage growth inhibition of 2.1, 16.0, 35.7, 41.0, 18.9 and 16.6, respectively, at a concentration of 3 µg/mL), evidencing some specificity of this type of compound on cell lines that overexpress P-gp [110].

### 2.6. Inhibitors from Marine Bacteria

The bacterium, *Nocardiopsis* sp. CMB-M0232, was isolated from a sediment sample collected in Australia. A non-saline liquid culture of this bacterium yielded two prenylated uncommon diketopiperazines (DKP): Nocardioazine A (**28**) (Figure 6) and nocardioazine B. Compound **28** reversed the resistance exhibited by SW620AD-300 cells equipotent to verapamil. Conversely, nocardioazine B showed no inhibitory activity against P-gp, which could mean that the strange-bridged DKP scaffold formed between one of the nitrogens, and the isoprene external unit might be involved in biological activity [111].

## 3. Conclusions

P-gp can expel a broad range of structurally different exogenous compounds out of the cells. For this reason, a very active P-gp transporter could potentially diminish drug delivery to the target organ and has been correlated to treatment resistance, despite peripheral drug concentrations that are within their therapeutic range.

Inhibition of P-gp leads to an increase in the permeability of some target organs. This result could permit administering lower drugs oral doses, and it may help to decrease drug toxicity. As a result, P-gp mediated drug efflux is recognized as a desirable target for therapeutic intervention in order to target and optimize the drug delivery of drugs to tumor cells and physiologically/anatomically isolated tissue compartments.

Marine organisms have proven to be an important source in the discovery and development of compounds that inhibit P-gp. Most of these compounds have demonstrated a reversal of multi-drug resistance in certain cancer cells. Importantly, these compounds have a potential application to be administered in conjunction with drug therapies that have been stymied by overexpressed P-gp, including anticancer agents, β-adrenoreceptor blockers, calcium channel blockers, cardiac glycosides, immunosuppressants, steroid hormones and antiparasitic, among others.

Importantly, the majority of the marine inhibitors outlined in the review had been scarcely studied, and very little is known about their mechanism of action. Extensive research, including that of a rational drug design to create derivatives with higher activity, less toxicity and less pharmacokinetic interactions, would lead to more clinically viable drug candidates. Nevertheless, all these agents represent new research tools for the discovery and development of efficient P-gp inhibitors, which may have potential use on their own or in combination with other therapeutic agents for the treatment of various diseases of clinical relevance.
